# Induced overexpression of MARCH-1 in human macrophages altered to M2 phenotype for suppressing inflammation process

**DOI:** 10.22038/IJBMS.2022.62893.13902

**Published:** 2022-04

**Authors:** Zivar Zangeneh, Gholamreza Khamisipour, Ali Reza Andalib

**Affiliations:** 1 Department of Immunology, School of Medicine, Isfahan University of Medical Sciences, Isfahan, Iran; 2 Department of Hematology, School of Para Medicine, Bushehr University of Medical Sciences, Bushehr, Iran; 3 National Institute of Genetic Engineering and Biotechnology (NIGEB), Tehran, Iran

**Keywords:** Inos, Macrophage, MARCH-1, Polarization, TGF-beta

## Abstract

**Objective(s):**

The M1 macrophage is characterized by enhanced pro-inflammatory cytokines production, whereas macrophage (M2) has anti-inflammatory features. Macrophage polarization as a therapeutic target for controlling immune responses could be performed by gene transduction to control the regulation of exaggerated innate/adaptive immune responses.

**Materials and Methods:**

Macrophages were prepared from THP-1 cell line and human monocytes that were transduced with (Membrane-Associated RING-CH-type finger) MARCH-1 viral lentivector produced in HEK-293T cells. RT-PCR and Western blotting confirmed MARCH-1 gene transduction. Cytokine production, CD markers assay, macrophage phagocytosis potential activity and mixed leukocyte reaction (MLR) with CFSE were performed for M1/M2 plasticity.

**Results:**

The mean fluorescent intensity of HLA-DR and CD64 expression reduced in MARCH-1+ transduced macrophage population. However, CD206 and CD163 expression increased in these macrophages. The concentrations of IL-6, TNF-α and iNOS were decreased in MARCH-1 transduced cells, and TGF-β production showed an augmentation in concentration. Western blotting and real-time PCR measurement confirmed that the expression levels of MARCH-1 protein and arginase-1 enzyme were increased in transduced macrophages.

**Conclusion:**

The anti-inﬂammatory features of MARCH-1 revealed the reduced levels of pro-inflammatory factors and maintained M2 phenotype characterized by high levels of scavenger receptors. Therefore, targeting MARCH-1 in monocytes/macrophages could represent a new autologous cell-based therapies strategy for inflammatory conditions.

## Introduction

Macrophages have polarized potential activity, which could act as coordinators in both innate and acquired immune responses. Macrophages involve through phagocytosis of apoptotic cells, neoplastic cells and microbes (1). In addition, by secreting cytokines/chemokines and interaction with T/B lymphocytes, macrophages support antigen elimination (2). Based on various stimulation in the environment around macrophage activation, two subsets of macrophages are classified, inflammatory or classical macrophage (M1-MQ) and anti-inflammatory or alternative macrophage (M2-MQ) (3). Inflammatory macrophages are activated by IFN-Ɣ, lipopolysacharid (LPS) of gram-negative bacteria and endogenous damaged-associated molecular patterns (DAMP) released from the necrotic or dying cells (3, 4). The properties of M1 are characterized by the expression of high levels of pro-inflammatory cytokines, including IL-1, IL-6, IL-12, TNF-α, IL-23, and inducible nitric oxide synthase (iNOS) that play a dominant role in Th1-mediated immunity (5, 6). However, the activity of anti-inflammatory macrophage (M2) begins in response to Th2-related cytokines such as IL-4 and IL-10, leading to the secretion of anti-inflammatory cytokines such as , IL-10 and TGF-β—they are involved in angiogenesis, tissue repair, and regeneration (7, 8). Inflammatory macrophage (M1) increases their expression of CD86, CD68, CD64 (FCƔR) molecules, while anti-inflammatory macrophage (M2) enhance their scavenger receptor (CD163) and mannose receptor (CD206) (9). Moreover, M2-MQ shows an increased amount in arginase-1 synthesis, but reduced iNOS production (1, 6). By changing the environmental stimuli, these two subclasses of macrophages could be converted to each other, which is called macrophage polarization (2). Due to diverse macrophage functions, the balance or plasticity between these two populations is essential for different pathological conditions. Numerous studies have reported macrophage polarization as a therapeutic target for controlling immune responses, especially in chronic inflammatory conditions (10). Therefore, by means of cytokines and chemokines treatment in changing the phenotype of inflammatory macrophage to non-inflammatory, as well as epigenetic manipulation such as changing in micro-nucleotide expression, transcription factor and post-translational modifications would be utilized for altering macrophage polarization (5, 11). Moreover, ubiquitinization process plays a central role in maintaining appropriate positioning of the protein , stability, and its interaction with other proteins within the cell (12). Ubiquitination is a multi-step process performed by different enzymes E1 (activating enzyme), E2 (conjugating enzyme), E3 (enzyme ubiquitin ligase). MARCH family protein (Membrane-Associated RING-CH-type finger) is a class of ubiquitin ligase enzymes that consists of 11 members (13). MARCH-I protein, a member of the ubiquitin ligase E3 family, is essential for expression in the immune cells and regulation of the immune response. MARCH-I expression is present in resting dendritic cells, naive B lymphocytes, and macrophages and monocytes though little (14). Primary immune response molecules targets for MARCH-I ubiquitination include major histocompatibility class II (MHC-II), CD86, TFR (Transferrin receptor), Fas (CD95), human leukocyte antigen (HLA-DM), HLA-DO, and CD98 (15). MARCH-I ubiquitinates a Lys residue on the MHC-II β-chain cytosolic domain and a cluster of Lys residues on CD86 (16, 17). As a result, the negative immunomodulatory role of MARCH-I is to inhibit dendritic cell maturation in antigen-presenting cells (18). MARCH-I expression is reduced by stimulation of dendritic cells and B lymphocytes by Toll-like receptor (TLR) ligands (18). Therefore, MARCH-I is known to inhibit dendritic cell maturation and antigen delivery to T lymphocytes. IL-10 is the only known inducer of MARCH-I in monocyte and macrophages (19). In MARCH-I^-/- ^mice, macrophage upregulates high levels of MHC-II molecule. The current study showed that MARCH-I controls the regulation of innate immune functions such as cell migration and inflammatory phenotypic properties of macrophages (19). In macrophages, some intracellular pathogens increase MARCH-I expression through IL-10 upregulation for their survival (20). In this case, MARCH-I contributes to pathogen survival by ubiquitinating CD95, a molecule associated with apoptosis. Therefore, ubiquitination CD95 by MARCH-I upregulation could improve macrophage survival. So, the present study was designed to evaluate induction and effectiveness of higher MARCH-I expression on phenotypic and functional changes in macrophage cell line as well as human monocyte-derived macrophages to help the polarization to M2 for suppressing inflammation in appropriate conditions.

## Materials and Methods

***Human MARCH-I gene information***

The DNA sequence of Human MARCH-I variant 2 (CCDS: 54814) has been verified against its coding sequence, in order to design and construct a recombinant vector containing the MARCH-I gene structure, from the NCBI reference sequence database (www.ncbi.nlm.nih.gov). Then, to increase the expression of the desired gene, the Kozak sequence (GCCACC) was considered at the 5´ end, and TAG sequence was considered at the 3´ end, for transcription termination. In the gene sequencing design, according to the cut site in the vector used, the BamH1 restriction enzyme cut sequence was placed at both ends of the gene sequence. The gene sequence with a final length of 870 bp was sent to Generay (China) for chemical synthesis based on the reference sequence.

***Cultivation and preparation of HEK-293T cells for plasmid transfection***

To produce lentiviral vectors, HEK-293T cells were cultured in T25 culture flask in high-glucose Dulbecco’s Modified Eagle Medium (DMEM) (Gibco, USA), supplemented with 10% fetal bovine serum (FBS) (Gibco, USA) and 100U/ml penicillin/streptomycin (Gibco, USA). The cells were transfected with PCDH1(The lentiviral packaging plasmids e.g., pPACKH1™ Packaging Plasmid mix), transfer plasmid containing MARCH-1 gene, PMD2.G envelope plasmid (an envelope protein VSV-G expressing plasmid pMD2G), and psPAX packaging plasmid (PAX2 plasmid is used for dendritic cells transduction, and psPAX seems to be common plasmid used for LV packaging) following the third passage, and when the cells reached confluences of 75-80%.,Two hours before the transfection, the medium was replaced with fresh medium containing 5% FBS. 

***Production of lentiviral vector***

 The plasmids PCDH-1 MARCH-1-GFP (21 μg/μl), psPAX2.221 (21 μg/μl), and PMD2.G (16.5 μg/μl) were dissolved in HBS (Hepes Buffered Salin) 2X buffer plus calcium chloride solution (CaCl2) for the transfection of the cells. The HEK-293T cells were then transferred into the T-25 flasks and were incubated at 37 °C with 5% CO_2_ incubator. Sixteen hours later, the culture medium was replaced with a fresh medium. The medium supernatant containing viral particles was collected 24, 48 and 72 hr post-transfection and replaced with a fresh medium. The culture medium containing the shedding virus was centrifuged (500 g, 10 min, at 4 °C) to remove the cell debris and filtered through an 0.45 μm filter. PEG8000 solution (50%) was added to the virus suspension by the final concentration of 5%, and 5 molar NaCl solution (5M) was added to the virus suspension to reach the final concentration of 1.5 M. Following that, the falcon tube was placed on the rotator for 24 hr at 4 °C to make a homogenous solution. Then, it was centrifuged at 4000g at 4 °C for 20 min. Finally, the pellet containing viruses was dissolved in 200 μl PBS and stored in a −70 ^o^C.

***Isolation of monocytes from buffy coat in blood bags***

Peripheral blood leukocytes were isolated from buffy coat in blood bags of healthy donors and washed twice with phosphate buffer saline (PBS) at 25 °C. Mononuclear cells were separated using Ficoll-Paque density gradient centrifugation (Baharafshan, Iran) at 1800g, 20 min, and broke off. The separated cells were cultured in RPMI1640 medium supplemented with 10% FBS and incubated at 37 °C and 5% CO_2_. After 2 hr, floating cells were removed from the surface. The cells adhering to the bottom of the flasks were considered monocytes. The purity of monocytes was evaluated using CD14+ marker by flow cytometry. 

***Macrophage development from THP-1 cell line and monocytes isolated from blood bag***

THP-1 cells were cultured in T25 flask in RPMI1640 medium, the cells were incubated with Phorbol-12-myristate 13-acetate (PMA) (Sigma-Aldrich) at a concentration of 50 ng/ml for 2 days to differentiate into macrophages. Then, the cells were nourished with a fresh culture medium without PMA for 24 hr. After evaluating the cells in terms of appearance and suitable morphology, they were ready for transduction. Monocytes isolated from the blood bag were incubated with Phorbol-12-myristate 13-acetate (PMA) (Sigma-Aldrich) at a concentration of 50ng/ml for 2 days to differentiate into macrophages.

***Macrophage transduction***

The macrophages derived from THP-1 cell line and the human monocytes were transduced with viral lentivirus produced from HEK-293T cells. The 3×10^5 ^macrophages were collected, mixed with 6ml of virus soup and 6µg/ml Polyberen, and the cell suspension was centrifuged at 500g, 30min, at 4 °C. Then, the cell pellet was dissolved in media (RPMI1640 with 10% FBS), cultured in each plate (12 well plate), and incubated at 37 °C, 5% CO_2_ for 14 hr. Fourteen hr post-transduction, the supernatant was replaced with media (RPMI1640 with 10% FBS). Forty-eight hours later, incubation was performed, and then light and fluorescent microscopes were used for examining the expression of MARCH-I protein fused to GFP inside the cells. In addition, the collected cells were prepared for flow cytometry. The following monoclonal antibodies were used: Anti-CD206-PE (clone GHI/61, cat no, CAT NO:333605, Biolegend), Anti-CD163-PE clone RM3/1, cat no, CAT NO: 323605, Biolegend, Anti-HLA-DR-PE clone L243, cat no, CAT NO:307605, Biolegend, Anti-CD64-PE clone 10.1, cat no, CAT NO: 983202, Biolegend, Anti-CD14-APC(clone-15-2, CAT NO: 321105, Biolegend). Notably,mean fluorescence intensity (MFI) for each marker was recorded separately. The flow cytometery data were analyzed using FlowJo software (version 7.6.2). The gating strategies were defined with the CD14+ and GFP+ subgroups. The GFP subset represents the expression of MARCH-1 to the GFP protein. PE Mouse IgG1K isotype control, (Clone: MOPC-31-C, BD Pharmingen), and Mouse IgG1K Isotype Control, (Clone: MOPC-21, BD, APC, USA) were applied for all the samples.

***Measurement of cytokines in culture medium supernatants***

The cytokine concentration measurement in macrophage culture supernatant after 72 hr of transduction using ELISA for TNF-α, IL-6, TGF-β and iNOS (all from R&D System, USA) was performed according to the kit manufacturer’s instructions.

***Western blotting***

For western blotting, the cells were lysed with RIPA buffer. The centrifugation was carried out at 14,000 rpm for 20 min at 4 °C. Protein concentration was determined by Bradford Protein Quantification Kit (Cat NO, DB0017, Kalazist, Iran) according to the manufacturer’s instructions. Cell lysates were mixed with an equal volume of 2X Laemmli buffer and boiled for 5 min. The SDS-PAGE analysis was then performed to separate the proteins. Then, the gel was transferred to an 0.2 μm immunoblotting membrane™ (polyvinylidene difluoride, PVDF) (Bio-Rad Laboratories, California, USA). The membranes were then blocked with 5% BSA (diluted in PBS-T) (Sigma Aldrich, MO, USA) with 0.1% Tween 20 for 1 hr. The membranes were then incubated with anti-β-actin antibodies against MARCH-1 (cat number: NBP1-59758, Novus Biologicals), and anti-ARG1 (cat number: NBP1-32731SS, Novus Biologicals) and incubated for 1 hr at room temperature. The membrane was then washed three times with TBST(Tris-buffered saline with 0.1% Tween 20 detergent), and incubated with goat anti-rabbit IgG H&L (HRP) secondary antibody (ab6721; Abcam)., Following that, the membrane was exposed to Enhanced Chemi-Luminescence (ECL) for 1-2 min. Protein expression was normalized to β-actin. Protein band densitometry was performed using gel analyzer software version 2010a (NIH, USA). In this way, band was considered as the percentage and compared with the corresponding β-actin band. Finally, the values were calculated and interpreted. 

***RNA extraction and cDNA synthesis***

The YTzol reagent (YTzol Pure RNA Cat No: YT9064) was used to extract RNA from 3x10^5 ^macrophage according to the manufacturer’s instructions. For cDNA synthesis from extracted RNA, a cDNA synthesis kit (Yekta Tajhis Azma- Iran) was employed based on the kit instructions. In the first step, 250 μl of the total RNA extracted from the macrophage was transferred to a sterile microtube. 0.2 µl of random hexamer primer and 1 µl of oligo-dT primer were added to the mixture. The final volume in the microtube was increased to 13.4 µl by adding DEPC water (Diethylpyrocarbonate-treated and sterile filtered water). The microtubes were then vigorously mixed and incubated at 70 °C (in a thermocycler) for 5 min. The microtube was then rapidly transferred on ice. After spinning the microtube, it was kept on the ice again. The next step was done according to the Table 1.

After adding the ingredients to the table, the microtube was first spined. Then, cDNA was synthesized at 37 °C for 5 min, and then at 70 °C for five minutes in a thermocycler and kept at − 70 °C .

***Real-time PCR***

Real-time PCR technique (RealQ Plus 2x Master Mix Green high Rox kit, Denmark) was applied to check the mRNA expression of MARCH-1 and housekeeping gene. The required materials are demonstrated in the following table. These primers were designed by gene runner software (as forward primer complementary to the vector backbone reverse primer complementary to a part of transgene) and purchased from Bonyakhteh, Iran. After gently mixing the above mixture, according to the program in the Table 2, it was done in the Real-time PCR machine (applied biosystem).

***Evaluation of macrophage phagocytosis potential activity***

To evaluate the potency of macrophage phagocytosis, Candida Albicans was cultured in maltose broth for 24 hr in 37 °C. In the next step, the tube containing yeast culture was centrifuged at 1000 g for 10 min to remove the culture medium. Then, the washing was performed two more times by a washing serum (PBS with FBS 10%). Finally, macrophages transduced with MARCH-1 gene were then treated with 200 ml (4×10^6^ yeasts per ml) of Candida. albicans yeast in RPMI1640 medium containing 5% FBS for 2 hr. After centrifugation, the supernatant was discarded, washed again with PBS buffer, and prepared from expanded cell precipitate. Then, it was stained with Wright-Geimsa dye solution. The results were evaluated using light microscopy for counting the percentage of yeast phagocytic macrophages compared to the control group (non-transduced macrophages).

***Staining of T lymphocytes with CFSE for considering mixed leukocyte reaction (MLR)***

The Jurkat cell line (T lymphocyte) was used to assess the proliferation of T lymphocytes in the presence of a MARCH-1 gene transduced macrophages. 2×10^5^ Jurkat cell suspension with CFSE dye (carboxyfluorescein succinidyl ester) with concentration (5 mM/l) in 100μl of complete RPMI1640 medium was added to 96 well plate wells. In addition, 2x10^5^ transduced macrophage cells were added to the above suspension in 100 μl of complete culture medium. The control wells are 2×10^5^ Jurkat cell suspension stained with CFSE in 100 μl of complete RPMI1640 culture medium. The other well contained non-transduced macrophages. Evaluation of T lymphocyte proliferation using flow cytometry in FL1 channel and the presence of CFSE dye is expressed as MFI. 

***Statistic analysis***

The obtained results were analyzed using “Mann-Whitney U” non-parametric test, and independent t-test where necessary. The *P*-value<0.05 was considered significant. The diagrams are drawn using Prism software.

## Results

***Producing lentivector containing MARCH-1 gene in HEK293T cell line, and its transduction to THP-1 cells and the macrophage derived from peripheral blood monocyte***

**Figure 1:**
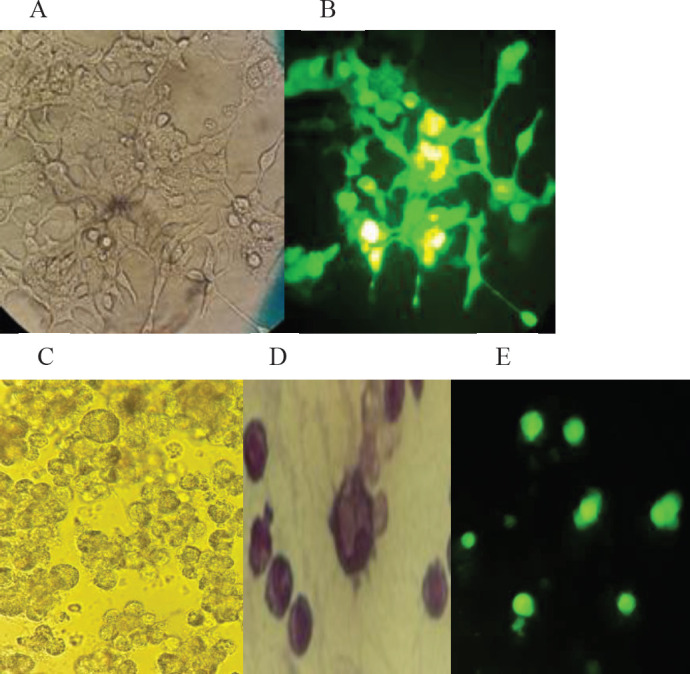
Indicate HEK293T cell post-transfection in tissue culture flask is compared with light (A) and fluorescent microscope observation (B) with magnification X40. Transduced Macrophage derived from THP-1 cells (with light (C) and fluorescent (E) microscope, Wright-Giemsa dye (D) are illustrated

Cultured HEK293T cell line with 75-80% confluency was transfected with the mentioned plasmids. Forty-eight hours post-transfection, observation of green fluorescent dye for GFP expression indicates transfection accuracy (Figure 1A, B). THP-1 cell line and the macrophage derived from peripheral blood monocyte were transduced with lentivector containing MARCH-1. The green fluorescent dye was seen 24 hr post-transduction (Figure 1C, D, and E) by the fluorescent microscope. The cell appearance was evaluated 24 hr post-transduction. Transduced macrophages with the MARCH-1 gene had less elongation in appearance than pre-transduction conditions and became spherical. The mean percentage of transduced macrophages were calculated 74±9 % in tissue culture, and MFI was calculated at 588±113% by flow cytometry analysis.

***CD marker assay in post-transduction cells by flow cytometry***

**Figure 2:**
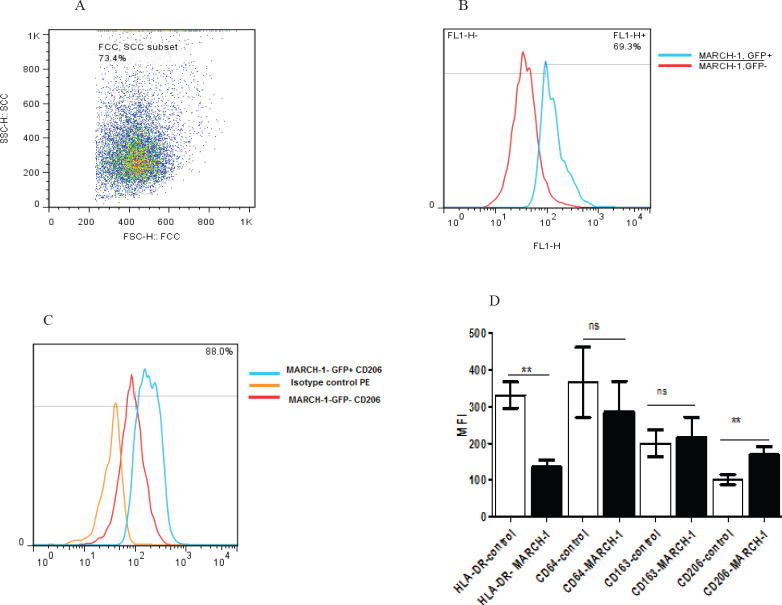
illustrates the dot plot representative sample of peripheral monocyte macrophage population by flow cytometry (A). The B illustrates percentage of macrophage population expressing GFP+ and non-transduced macrophages. The C plot is histogram of isotype control and CD206 in the two mentioned groups. The D section indicates MFI of HLA-DR, CD206, CD163, CD64 markers in GFP+ (black column) and GFP-macrophages (white column). The data is shown as mean±SD

The flow cytometry analysis showed that the MARCH-1GFP+ expressing peripheral monocyte-derived macrophage population increased by 69.3% compared to non-transduced population (Figure 2B). The transduction of MARCH-1 in the macrophages caused alteration in HLA-DR, CD64, CD206 and CD163 marker expression. The mean fluorescent intensity (MFI) of HLA-DR expression in MARCH-1+ macrophage population reduced from 331±35 to 134±19 arbitrary unit (*P*=0.008) and CD64 decreased from 366±95 to 284±85 arbitrary unit as well (*P*=0.015). However, CD206 expression in transduced macrophages rose from 100.6±13.7 to 168±22 arbitrary unit (*P*=0.008). The MFI for CD163 expression in transduced macrophages was 215±55 compared with the non-transduced cells with 199.8±85 arbitrary unit (*P*=0.54) (Figure 2D). These data are taken from the experiments in both human cell-derived macrophages and the cell line, and due to the similarity of the means, the human macrophage data are presented. In cell-derived macrophages, two measurements were performed and similar results were obtained with human macrophages.

***Cytokine production in MARCH-1 transduced macrophages***

The cytokine concentration of IL-6, TNF-α, iNOS and TGF-β production was assayed in the supernatant of culture medium for both THP-1 cells and macrophages after transduction process with MARCH-1. The concentration of IL-6, TNF-α and iNOS was lower than the non-MARCH transduced cells after 48-72 hr of culture. However, TGF-β production showed an augmentation in concentration assay in the supernatants 72 hr after transduction. The measurement data are shown in Table 3.

***Western blot analysis***

The expression levels of MARCH-1 protein and arginase-1 enzyme were assayed by Bradford Protein Quantification technique for transduced and non-transduced cells. After performing protein band densitometry, the data were obtained and arbitrary units for comparing the protein density in the gels are shown in Figure 4A, B. The arbitrary units and relative percentages are shown in Table 2 for comparing the protein expression potentials after the gene was transduced in the cells. The increase for MARCH-1 gene expression in transduced macrophages was approximately double compared to the non-transduced macrophage after 72 hr after transduction. Arginase-1 production was wnhanced from 41% based line level to 67% compared to β-actin production (Table 4).

***Real time PCR measurement***

The expression of MARCH-1 gene in peripheral monocyte-derived macrophage transduced with MARCH-1 was measured using real-time PCR, and the results are shown in Figure 5.

***Macrophage phagocytosis activity***

Increased expression of MARCH-1 gene in vector-transduced macrophage reduces the phagocytic potential activity for phagocytosis of yeast. A total of 200 μl of yeast solution concentration (4×10^6^ mg/ml) was mixed with 1000 macrophages for 2 hr. The number of phagocyted yeasts was counted in the macrophages. In addition, the data obtained shows that the percentage of phagocytic cells for the transduced cells was 76% and for non-transduced cells it was 82% (Figure 6).

***Mixed lymphocyte reaction with MARCH-1 transduced macrophages***

The amount and intensity of CFSE fluorescent were evaluated in the three co-culture groups of 1. Jurkat T cell line and transduced macrophage with MARCH-1 gene;.2. Jurkat T cells and non-transduced macrophage; and 3.Jurkat T cell line alone. The intensity of fluorescent dye in the macrophage group (group 1) derived from peripheral blood monocytes transduced with the MARCH-1 gene (A) was obtained 81% and in the non-transduced macrophage it was calculated at 76% (B). These data analyses did not show significant differences between group 1 and 2 (*P*=0.068) (Figure 7). 

**Table 1:** RNA extracted from transduced macrophage demonstrated below

5x first-strand buffer	4L
dNTPs (10 mM each) ‌	1L
RNase in (40U/μl) ‌	0.5L
M-MLV	1L

**Table 2:** MARCH-1 gene expression are demstrated below

Quantity L	Material	Quantity L
7.5 L	Master Mix Green with high ROX™	7.5 L
1 L	Forward primer TTGGCTTCACAGGAGGTGTT	1 L
1 L	Reverse primer GCACTACCACAGCATCTTTGA	1 L
1 L	cDNA	1 L
3 L	Distilled water	3 L
13.5 L	Total volume	13.5 L

**Table 3.** The cytokine concentration in supernatant culture medium of transduced cells with MARCH-1 compared with the non-transduced cells

*P*-value	MARCH-1 transduced Macrophage derived primary monocyte	non-transduced (Macrophages)	*P*-value	MARCH-1 transduced (THP-1 cells)	Non-transduced (THP-1 cells)	Cells
						Cytokines
0.03	16.281.89	20.071.84	0.02	12.281.7	17.43 1.77	IL-6
0.04	21.58.9	33.846.22	0.04	34.447.77	45.838.82	TNF-
0.04	15.83.45	23.0 4.4	0.03	11.694.48	19.0 3.4	iNOS
0.002	79.038.21	34.56 4.01	0.003	82.687.2	41.56 8.01	TGF-

**Figure 3:**
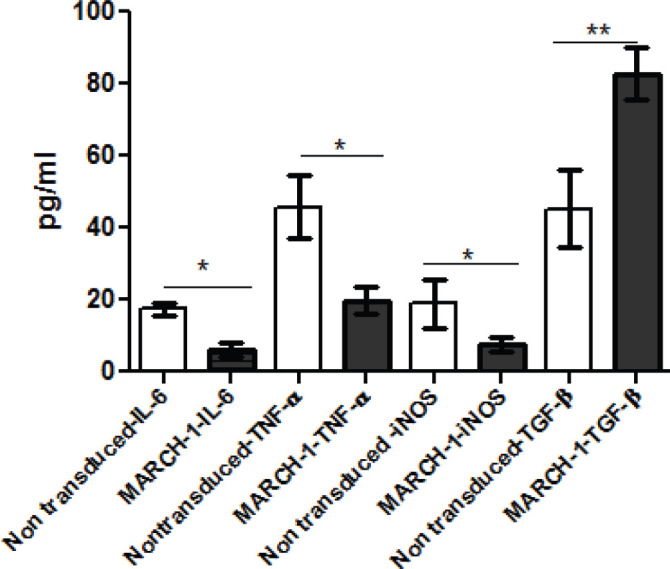
Comparing the concentration of the cytokines measurement in cell culture supernatants in the two groups of MARCH-1 transduced macrophages and the non-transduced macrophages

**Figure 4:**
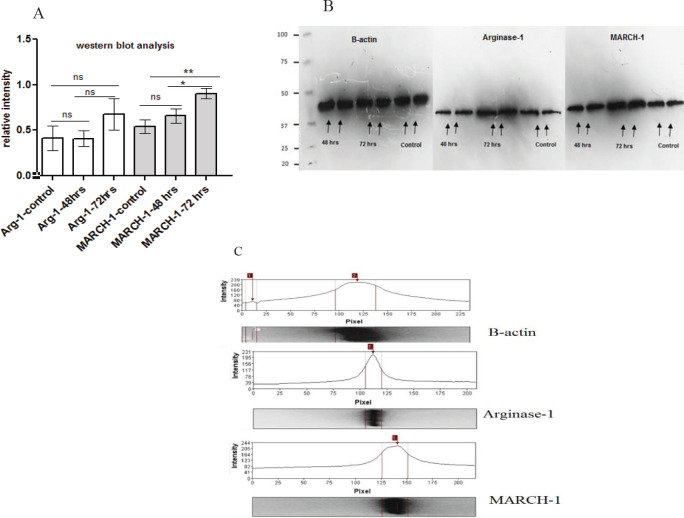
Western blot analysis and gel protein densitometry were used for quantification of MARCH-1 and Arginase-1 proteins on transduced macrophage D: The gel image indicates the blot results 48 and 72 hr after transduction, compared with the non-transduced cells

**Table 4:** Western blot relative densitometry data with arbitrary units and relative percentages for transduced cells

MARCH-1 production	Arginase-1 production	-actin production	
4577 411	3448 110.1	8457129.4	Non transduced cells
55% 13	41% 3	100%	Relative Intensity
5805109	3649 78	880768.59	48hrs after transduction
66% 10	40%5	100%	Relative Intensity
7711 307.1	5738 259	8515268.0	72hrs after transduction
91% 8	67%10	100%	Relative Intensity

Data were calculated as mean ± standard deviation for the five samples. The arbitrary units were used in densitometry

**Figure 5:**
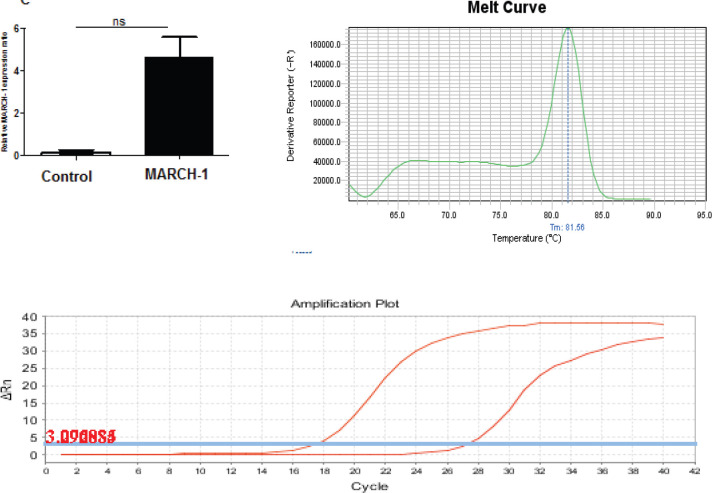
The ratio of increased expression of MARCH-1 gene to HPRT is shown in the non transduced macrophage (control) and transduced macrophage. MARCH-1 gene in transduced macrophages was 5.3-folds expression compared to the non-transduced macrophage (*P*<0.0034). Data were calculated as mean ±standard deviation

**Figure 6:**
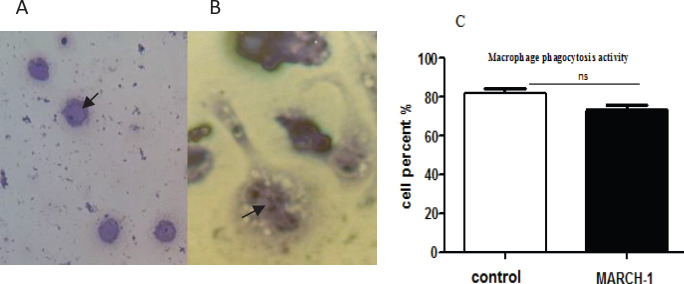
Phagocytosis activity assessment for transduced macrophages by Wright-Giemsa dye

**Figure 7:**
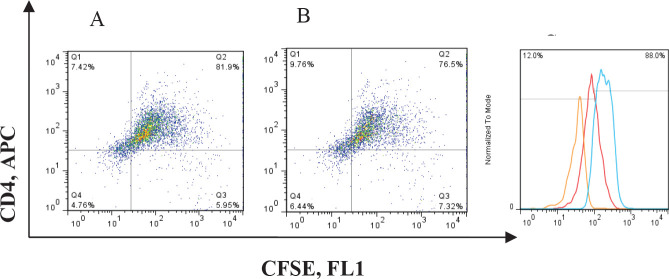
A and B; the T lymphocytes plot selected based on the CD3 and CD4 markers after proximity to the THP-1 macrophage cell line. C: Lymphocyte proliferation rate in the blue histogram: Jurkat T lymphocyte without macrophage proximity. Red: Jurkat T lymphocyte adjacent to the non-transduced macrophage. Orange: Jurkat T lymphocyte adjacent to human macrophage transduced with MARCH-1 gene

## Discussion

Macrophages, as part of the innate immune response, are phagocytic cells which play a central role in both infectious and non-infectious circumstances (21). The plasticity feature of heterogeneous macrophages allows the optimization of their phenotype and functions in response to rapid changes in the surrounding microenvironment, such as infectious pathogens (22). In recent studies according to clinical trials, administration of macrophages did not show adverse events; therefore, monocyte/macrophage-based therapies with high level of safety is recommended (23). Despite that, the main limitation for the effectiveness of macrophage-based therapies is great plasticity features of this cell in that they quickly change their phenotype and function depending upon micro-environmental stimuli (4). Genetic modifications by vectors and microRNAs due to long-standing gene expression are novel pathways to overcome this limitation for macrophage cell therapies. According to this, Michael *et al*. reported that CAR­Macrophage (Chimeric antigen receptor, CAR) after systemic administration persisted for at least 62 days *in vivo *(4). The main goal of cell therapy is safety for patients; therefore, autologous modified cell transplant is the best choice. For this reason, in our study, the phenotypical and functional impacts of MARCH-1 on both human primary macrophage and THP-1 cell line by lentivector transduction were investigated. However, gene transfer into primary human macrophages has been a longstanding challenge, in this study, overexpressed MARCH-I macrophage was produced after 48 hr post-transduction and maintained for 9 days in the culture medium with M2 phenotype (alternative macrophage) *in vitro*. Our data indicate M2 phenotype in macrophage due to transduction with MARCH-1 which significantly reduced MHC-II (HLA-DR) and CD64 (FCγR1, as a biomarker of inflammatory macrophage) expression on macrophage. Schriek *et al*. reported that in human monocyte-derived DCs, mouse DCs and B cells, peptide-loaded MHC-II (HLA-DR) molecules reduced their stability at the cell surface and increased internalization due to ubiquitination by MARCH-1 (24). In our research lab, transduced dendritic cells with MARCH-1 reduced their surface expression of HLA-DR on the cells. These cells show immunosuppressive effect on human T lymphocyte that could increase regulatory T cells. The results of this study suggest that transduced macrophage with MARCH-1 can maintain anti-inflammatory phenotype over time with considerable potential as an autologous treatment cell therapy strategy across a wide range of pathological conditions (23, 25). Further studies demonstrate that MHC-II is less polyubiquitinated and more firmly expressed on the cell surface in mature DCs and B cells in MARCH-I−/− compared with wild-type mice, resulting in enhanced antigen-presenting ability (16). Mittal *et al*. explained tolerogenic activity of regulatory T cells (Treg) and IL-10 mediated through MARCH-I up-regulation. It promotes surface MHC-II and CD86 internalization in human primary monocytes and macrophages and dampens the innate inﬂammatory response in mice (20). The anti-inﬂammatory features of MARCH-1 revealed reduced expression of pro-inﬂammatory cytokines including IL-6 and TNF-α and reduced expression of iNOS, too. Galbas *et al*. showed that MARCH-I^-/-^ conventional dendritic cells have increase in the systemic production of proinflammatory cytokines, especially IFN-¥. The results from our study, could be explained the strengthen anti-inflammatory roles of some cytokines by MARCH-I ability induction. In addition, it was found, in our study, that MARCH-1 overexpression on macrophages leads to a significantly elevated level of TGF-β expression. Altogether, the reduced levels of pro-inflammatory factors (IL-6, TNF-α and iNOS) along with the elevated level of TGF-β due to MARCH-I overexpression result in a maintained M2 phenotype. We conclude that gene transfer with MARCH-1 could help cell transplantation for a long time *in vivo *because this gene controls macrophage phenotype and plasticity by multiple parameters, including decreased inflammatory markers: HLA-DR, CD64, IL-6, TNF-α and iNOS accompanied with elevated TGF-β. In addition, the effect of MARCH-1 overexpression resulted in an enhanced expression of CD163 and CD206(26). M2 Macrophages are characterized by high levels of scavenger receptors like CD163 and CD206 (26). Expression of CD163 by monocytes and macrophages serves as both a lineage-speciﬁc cell marker and a functional pattern recognition receptor with key roles in immune regulation (27). CD163 directly mediates the binding and uptake of hemoglobin complexed with haptoglobin limiting oxidative tissue damage. CD163 can also clear damage-associated molecular patterns, such as endogenous high-mobility group box 1 (HMGB1), and directly trigger anti-inﬂammatory functions of tissue macrophages (9). Yingxia *et al*. showed that Genistein reduced the expression of CD163, p-STAT3 and the levels of IL-10, increased the levels of IL-12 and nitric oxide in THP-1 macrophages, indicating that GEN can reverse M2 polarization of THP-1 macrophages (28). We evaluated MARCH-1 upregulation to induce Arginase-1 protein expression. Induction of Arginase-1 is considered a hallmark of M2 polarization in humans and mice (11) and we conﬁrmed in this study an enhanced protein expression of Arginase-1 following MARCH-1 up-regulation. Arginase-1 catalyzes the hydrolysis of arginine to ornithine, resulting in increased polyamine synthesis, which has an important role in tissue repair and remodeling (29). Our data show that macrophages with MARCH-1 overexpression had similar phogocytosis ability compared to the non-transduce macrophages. We finally evaluated the effect of macrophages with MARCH-1 overexpression on lymphocyte T cell line proliferation by MLR test. These data represented that MARCH-1 did not affect the cell expansion and proliferation with T cell line after exposure to macrophages with MARCH-1 overexpression. 

## Conclusion

In this study, we found that increased expression of MARCH-1 in monocytes/ macrophages could be responsible for macrophage polarization to M2 macrophages. Thus, targeting MARCH-1 in monocytes/macrophages could represent a new autologous cell-based therapies strategy for inducing anti-inflammatory conditions such as cerebral palsy, arthrosynovitis, diabetic wound, atherosclerosis and so on. Clinical experiments would be necessary for testing the effect of M2 macrophage population to assay reduction of the inflammatory factors. 

## Authors’ Contributions

ARA and GhRKh Conceived and designed the study ZZ, Performed data processing and collection, experiments, analysis and interpretation of results, draft manuscript preparation, and visualization; GhRKh and ARA Critically revised and edited the article; ARA Approved the final version to be published; ARA Supervised and helped with funding acquisition.

## Conflicts of Interest

The authors declare that there are no conflicts of interests.
